# Time series anomaly detection in helpline call trends for early detection of COVID-19 spread across Sweden, 2020

**DOI:** 10.1038/s41598-025-20641-2

**Published:** 2025-09-24

**Authors:** Atiye Sadat Hashemi, Dominik Dietler, Tove Fall, Malin Inghammar, Anders F. Johansson, Carl Bonander, Mattias Ohlsson, Jonas Björk

**Affiliations:** 1 Division of Occupational and Environmental Medicine, Department of Laboratory Medicine, Faculty of Medicine, Lund University, Lund, Sweden; 2 Molecular Epidemiology, Department of Medical Sciences, Uppsala University, Uppsala, Sweden; 3 Section for Infection Medicine, Department of Clinical Sciences Lund, Lund University, Lund, Sweden; 4 Department of Hospital Hygiene and Infection Prevention and Control, Lund, Region Skåne Sweden; 5 Department of Clinical Microbiology and Molecular Infection Medicine, Umeå University, Umeå, Sweden; 6 School of Public Health and Community Medicine, Institute of Medicine, Center for Societal Risk Research, University of Gothenburg, Karlstad University, Karlstad, Sweden; 7 Center for Environmental and Climate Science (CEC), Faculty of Science, Lund University, Lund, Sweden; 8 Clinical Studies Sweden, Forum South, Skåne University Hospital, Lund, Sweden

**Keywords:** Anomaly detection, Community spread, COVID-19, Early detection, Helpline calls, Symptoms, Infectious diseases, Epidemiology

## Abstract

Timely detection and surveillance of disease community spread is a potent tool for implementing effective public health interventions. This study investigates the National Telehealth Service (1177 helpline) across 18 regions in Sweden in 2020 to identify early signals of community transmission of COVID-19 at the beginning of the pandemic. Focusing on calls related to key COVID-19 symptoms (cough, fever, and breathing difficulties in adults), we analyze their frequency and distribution across referral categories, comparing them to 2019 data. We employ an explainable time series anomaly detection algorithm using daily call data to identify the first collective anomalies across regions. The results show that anomalies in call data were correlated with, but preceded, the first confirmed case infected in Sweden by a median of 7 days (IQR: 2.5–10.5) and the first hospitalized case infected in Sweden by a median of 13 days (IQR: 7.25–16). They also preceded the estimated onset of community spread, indicated by the absolute confirmed cases (median: 24.5, IQR: 18.25-32.5), and severe outcomes defined by hospitalizations (median: 33, IQR: 27.25-44). These findings showcase how helpline call monitoring, using time series anomaly detection, can aid early outbreak detection.

## Introduction

The coronavirus disease 2019 (COVID-19), caused by severe acute respiratory syndrome coronavirus 2 (SARS-CoV-2), was first identified in December 2019 in Wuhan, China^1^. Its rapid spread across Asia, Europe, and the rest of the world led to a global pandemic declaration by the World Health Organization in March 2020^2^. Europe experienced its first wave of infections in early 2020. In Sweden, the first SARS-CoV-2 infection was detected on January 31^3^, a traveler from Jönköping returning from Wuhan, China^4^. In March 2020, community transmission was evident, posing significant challenges for public health surveillance and containment^5,6^.

Surveillance of community spread is essential for effective public health responses^6^. Various approaches exist for disease surveillance, with one method being the monitoring of confirmed cases through widespread testing programs^7^. However, case counts are an unreliable metric during the emergence of a novel infectious disease due to limited testing, lab capacity, and selection bias in severity and demographics, hindering accurate outbreak tracking and decision-making. Alternative approaches, such as monitoring syndromic health data, can provide near-real-time insights into emerging diseases, as evidenced by research conducted in the United States and the United Kingdom^8–11^. Other surveillance approaches include wastewater sampling in the United States^12^, app-based symptom reporting in Sweden^13^, England and the UK^14–17^, and search ranks from internet search engines in the United States^18^.

Patients infected with the SARS-CoV-2 virus may experience symptoms such as cough, fever, breathing difficulties, sore throat, diarrhea, headache, muscle or joint pain, fatigue, and loss of smell and taste^19^. In Sweden, data on such symptoms can be gathered from the National Telehealth Service (1177 helpline)^20^. This telehealth service for triage and referral is operated by registered nurses and is available for everyone free of charge^21,22^. It is also widely used among various population groups, including the elderly population^23^.

The call data from the 1177 helpline has been analyzed for its potential use in the national surveillance of various diseases in Sweden^24–27^. For instance, Bjelkmar et al.^24^ demonstrated the early detection of a large waterborne cryptosporidiosis outbreak in Skellefteå by retrospectively linking calls to the 1177 helpline with water distribution areas. Martin et al.^25^ introduced a novel approach to tick-borne encephalitis (TBE) surveillance, utilizing data from the 1177 helpline, diagnosed case reports, and environmental factors to improve the detection and monitoring of TBE outbreaks between 2010 and 2017. Ma et al.^26^ assessed the effectiveness of three syndromic surveillance methods, web queries, 1177 helpline data, and school absenteeism, in detecting influenza activity in Sweden. Their findings revealed that web queries and 1177 helpline data produced results comparable to traditional surveillance systems, while school absenteeism data proved unreliable. Although these tools were not consistently early indicators, they could detect influenza cases before primary healthcare systems. Andersson et al.^27^ also compared local outbreak signals from 1177 helpline, web queries, and OTC antidiarrheal sales against known outbreaks, identifying helpline data as the most effective tool for early detection and monitoring. Some spatiotemporal analyses have also been conducted for COVID-19 in Sweden or for specific regions^28–30^.

The purpose of our study is to investigate whether the 1177 helpline in Sweden could serve as an early indicator of emerging disease patterns during the COVID-19 pandemic. The specific aim is to test the hypothesis that collective anomalies, groups of data points exhibiting abnormal behavior, in 1177 helpline call patterns could provide early signals of key pandemic outcomes, such as the onset of community spread and severe cases leading to hospitalization or death. To address this, we develop an algorithm to detect anomalies in time series call data and visualize their spatiotemporal patterns. We also conduct a comparative analysis of 1177 call patterns from the early phase of the COVID-19 pandemic in 2020 and the corresponding period in 2019, focusing on COVID-19 key symptoms, and examining their frequency and distribution across referral categories. Using the COVID-19 pandemic as a case study, this approach aims to enhance syndromic surveillance methods for detecting emerging disease patterns in future outbreaks.

## Telehealth call and COVID-19 data

Telehealth call data in this study includes records from the 1177 helpline, which provides medical counselling on care and illnesses in Sweden^20,31^. Registered nurses who answer calls on the 1177 helpline use a medical decision support system to assess the healthcare needs of callers^32^. Each call is classified according to one specific reason from a predetermined list (known as Contact Reason). We also utilize the public data on SARS-CoV-2 confirmed case from Sweden’s public health authority (based on the SMINET register)^33^. Furthermore, the individual-level data from 1177 helpline calls is linked with hospitalization data (National Patient Register, Inpatient and Outpatient Care) and death data (Cause of Death Register) from the Swedish National Board of Health and Welfare, to analyze community spread. The SARS-CoV-2 confirmed case, hospitalization, and death data sources cover the entire country and include individuals of all age groups during the study period.

Table 1 shows the overview of the 1177 helpline dataset. A key variable in this study is ”Contact Reason”, encompassing about 190 distinct codes for reasons ranging from common symptoms such as cough, fever, and abdominal pain, to sleep problems as well as for general medical inquires or administrative matters (e.g., scheduling appointments). In the dataset, only one main ”Contact Reason” is recorded per call, reflecting the primary reason for contact as assessed by the nurse. Specifically, our analysis focuses on three key COVID-19 symptoms i.e., cough, fever, and breathing difficulties by adults^34^ to elucidate changes in their combined frequency, distribution, and temporal trends. Another key variable, ”Referral Priority”, includes five unique categories: Immediate, Urgent, Within the next 24 hours, Next weekday, and Wait. For analysis, these categories are grouped into Emergency care (Immediate, Urgent), Primary care (Within 24 hours, Next weekday), and Wait. A Norwegian study shows that callers understand the advice given by registered nurses, and the large majority of patients advised to wait did not contact their GP or other healthcare services again with the same complaints the following week^35^. This suggests the classification of calls based on the ”Contact Reason” and ”Referral Priority” variables appear to be instrumental in managing healthcare needs efficiently and ensuring appropriate care pathways^35^.

**Table 1:** Overview of the 1177 helpline dataset in this study.

Variables	Unique in dataset	In this study
Contact reason (symptom)	$\sim$ 190 symptoms (including physical and mental related symptoms)	- Cough - adult - Fever - adult - Breathing difficulties - adult
Referral Priority	- Immediate (usually by ambulance to the emergency department) - Urgent (seek care at the emergency department) - Within the next 24 hours (seek emergency time at primary care or doctor on call) - Next weekday (primary care within a week) - Wait	- Emergency care (Immediate, Urgent) - Primary care (Next weekday, Within the next 24 hour) - Wait (Wait)
Spatial	21 regions in Sweden: Stockholm (R1), Uppsala (R3), Södermanland (R4), Östergötland (R5), Jönköpings län (R6), Kronoberg (R7), Kalmar (R8), Gotland (R9), Blekinge (R10), Skåne (R12), Halland (R13), Västra Götaland (R14), Värmland (R17), Örebro (R18), Västmanland (R19), Dalarnas (R20), Gävleborg (R21), Västernorrland (R22), Jämtland-Härjedalen (R23), Västerbotten (R24), Norrbotten (R25)	Analysis of symptoms: 18 out of 21 regions in Sweden (all regions excluding Stockholm (R1), Östergötland (R5), and Gotland (R9))
Temporal	Granularity: time stamp of individual calls	Granularity: daily aggregation From 01.01.2019 to 31.12.2020

The dataset spans January 1, 2019, to December 31, 2020, capturing COVID-19-related activity and comparing helpline calls before the pandemic and during its first year.

The geographical coverage of our call data includes all regions in Sweden, excluding Stockholm, Östergötland, and Gotland, since data were not available. Specifically, we consider data from 16 regions, excluding the regions of Värmland and Södermanland from January 2019 to October/November 2019, and from 18 regions thereafter (because Värmland and Södermanland began using the 1177 system through Inera in October/November 2019). We present call data figures relative to regional population size. For reference, the populations of the regions in 2019 are listed in Table 2^36^.

## Method development

In this section, we outline the approach for detecting the first collective anomalies in symptoms reported to the 1177 helpline, which are then visualized through geographic mapping to highlight regional patterns. Additionally, we present the estimation of the onset of community spread and severe outcomes based on data from confirmed SARS-CoV-2 infections, hospitalizations, and deaths.

### Detecting first anomalies in call data

Time series anomaly detection models are used in real-time surveillance systems, with different types of anomalies that can be prioritized depending on the specific application^37^. In time series data, anomalies can be classified into various types, including point-wise, contextual, and collective anomalies^38^. Point-wise anomalies refer to individual data points that deviate significantly from the overall distribution, while contextual anomalies arise when a data point deviates from expected behavior in a specific context. Collective anomalies, on the other hand, involve a group of data points that together exhibit abnormal behavior. Detecting collective anomalies requires examining the relationships between consecutive data points over time. While it is challenging to isolate specific types of anomalies as they are often interrelated, we aim to place a stronger emphasis on detecting collective anomalies. This is because sustained patterns over time, such as consecutive days of high call volumes to a helpline, are more likely to indicate meaningful trends rather than isolated anomalies.

To detect the first collective anomaly, we propose an algorithm that analyzes daily call signals derived from observed data in each region. The algorithm accounts for weekly and collective-term patterns through two key conditions:

- Weekly Average Condition: which evaluates whether the current day’s value exceeds the 7-day rolling average from the previous week by a dynamic threshold.
- Sequential Anomaly Condition: which identifies sustained increases over 1 to 7 consecutive days by comparing each sequence of days to the corresponding sequence from the previous week. It accounts for fluctuations in call volume that vary by weekday and requires the current sequence to exceed its past counterpart by a dynamic, proportional threshold, prioritizing collective anomalies over isolated spikes.

Mathematically, the applied algorithm can be expressed as:

1 $$\begin{aligned} (X_t> \alpha _t \cdot \bar{X}_{\text {last\_week}}) \quad \text {and} \quad \bigvee _{k=1}^{7} \left( \bigwedge _{i=0}^{k-1} \left( X_{t-i} > \left( 1+\frac{\alpha _t}{k} \right) X_{t-7-i} \right) \right) \end{aligned}$$

where $X_t$ denotes the observed data (in this study, it is the sum of calls for the three specified COVID-19 symptoms) in a specific region on day $t$, $\alpha _t$ is a dynamic threshold, and *k* represents the number of consecutive recent days (ranging from 1 to 7) being evaluated for sequential anomalies compared to the corresponding days in the previous week. The equation detects anomalies by evaluating the current value against two contextual conditions as mentioned above. First, it compares $X_t$ with the 7-day average ($\bar{X}_{\text {last\_week}} = \frac{1}{7} \sum _{i=1}^{7} X_{t-i}$). Then, it checks the sequential anomalies over varying sequence lengths to verify if the values over any 1 to 7-day period are significantly larger than the corresponding values from the previous week, capturing sustained and sequential increases.

We employ a dynamic threshold for the parameter $\alpha _t$ in Equation (1) to improve sensitivity and reduce false positives. Rather than using a fixed value, $\alpha _t$ is adjusted over time according to the rolling standard deviation of the data within a 7-day window. This dynamic threshold is expressed as $\alpha _t = \alpha _0 + c\sigma _t$ where $\alpha _t$ represents the threshold at time $t$, $\alpha _0$ is the base threshold value reflecting the initial sensitivity to anomalies determined from the data, $\sigma _t$ is the rolling standard deviation of the data over the specified window, and $c$ is a scaling factor that adjusts the contribution of $\sigma _t$, which in this study is set to 0.001 after tuning using random search. To ensure generalizability and avoid overfitting to region-specific patterns, the algorithm and parameters, including $\alpha _0$ and $c$, were applied uniformly across all regions without any region-specific optimization.

Seasonal factors such as school breaks, national holidays, and vacation periods can influence telehealth call volumes and healthcare-seeking behavior. While the main analysis did not explicitly adjust for seasonality in order to avoid excluding meaningful anomalies coinciding with seasonal patterns, we additionally implemented a complementary seasonality adjustment as:

2 $$\begin{aligned} (X_t> \alpha _t \cdot \bar{X}_{\text {last\_week}}) \quad \text {and} \quad \bigvee _{k=1}^{7} \left( \bigwedge _{i=0}^{k-1} \left( X_{t-i}> \left( 1+\frac{\alpha _t}{k} \right) X_{t-7-i} \right) \right) \text{and} \quad \bigvee _{k=1}^{7} \left( \bigwedge _{i=0}^{k-1} \left( X_{t-i} > \left( 1+\frac{\alpha _t}{k} \right) X_{t-i-365} \right) \right) \end{aligned}$$

where the last condition aims to check the current call volumes to the same period in the previous year. This approach balances sensitivity to both seasonal and outbreak-driven changes in call patterns.

While the proposed method focuses on developing an explainable data-driven algorithm for detecting collective anomalies, we also employ a z-score-based anomaly detector^39^ to identify significant temporal deviations, providing a baseline for comparison. This method uses a 30-day rolling window to calculate the mean and standard deviation. The z-score is computed by subtracting the rolling mean from the current value and dividing by the rolling standard deviation. Anomalies are flagged when the z-score exceeds a predefined threshold, indicating a significant deviation from the mean. Due to the data’s high variability, an appropriate threshold was selected to balance sensitivity and specificity. The first anomaly date for each region is recorded and presented in the results for comparison with our algorithm.

### Community spread and severe outcomes: estimation of date of onset

To assess the temporal relationship between anomalies in call data and community spread, we first identify key event dates for each region. As community spread refers to widespread disease transmission without identifiable sources like travel, we report the first COVID-19 confirmed and hospitalized cases (ICD-10 codes U07.1 and U07.2) infected in Sweden for each region, as shown in Table 2. We also provide additional dates, including the first confirmed and hospitalized cases, and the first death, regardless of the infection source (might be linked to travel) in each region. It should be noted that although the first case in Sweden was reported in the media on January 31 in Jönköping, in our dataset, it is registered on February 4 in the same location. Moreover, COVID-19 hospitalization or death in a region markedly before the first confirmed case were regarded as registration errors and thus removed (three cases in Stockholm and one case in Skåne).

As a complementary approach, we also estimated the date of onset of community spread based on the regional data, including the daily number of SARS-CoV-2 infections confirmed cases and the onset of severe outcomes based on hospitalization, and death.

Specifically, we define the onset of community spread in a region as the point when the cumulative number of confirmed cases, hospitalizations, or deaths caused by COVID-19 reaches a specified threshold for that region. In this study, we apply relative and absolute thresholds based on regional population sizes to define community spread, using data from 2020. The relative threshold for defining significant outbreak activity is set at 25 confirmed cases per 100,000 people (cumulative over the entire pandemic)^40^. Alternatively, an absolute threshold is also calculated based on the median population size of regions, i.e., Gävleborg (287,382). Applying the same rate (25/100,000) to this population yields 75 cases, rounded upward, as the absolute threshold. Thresholds for hospitalizations and deaths follow the same logic, with relative and absolute thresholds set accordingly.

In the Results section, we present trend analyses to examine changes in calls to the 1177 helpline before the pandemic (2019) and after its onset (2020), along with a referral priority analysis to evaluate shifts in nurse-assigned urgency levels over the same period. We also assess the correlation between the timing of the first anomalies in call data and the onset of community spread and severe outcomes, using Spearman’s rank correlation coefficients. Additionally, we report the median difference between the first anomaly and key public health milestones.

**Table 2:** Summary table for the important dates and statistical analysis of first detected anomalies in call data compared to other important dates in Sweden in 2020.

Region	Population (2019)	First anomaly in call data ^1^	First confirmed case	First confirmed case infected in Sweden	First hospitalized case	First hospitalized case infected in Sweden	First death
Stockholm (R1)	2,377,081	–	Feb 27	Feb 28	Feb 27	Feb 28	March 07
Uppsala (R3)	383,713	Feb 27	Feb 28	March 11	Feb 27	March 16	March 20
Södermanland (R4)	297,540	March 11	March 09	March 13	Feb 28	March 15	March 16
Östergötland (R5)	465,495	–	March 12	March 13	March 09	March 13	March 19
Jönköpings län (R6)	363,599	March 01	Feb 04	March 09	March 12	March 09	March 24
Kronoberg (R7)	201,469	March 05	March 10	March 14	March 23	March 25	April 05
Kalmar (R8)	245,446	March 11	March 10	March 18	March 10	March 18	March 26
Gotland (R9)	59,686	–	March 11	March 17	March 26	March 26	April 08
Blekinge (R10)	159,606	March 11	March 10	March 10	March 26	March 27	March 26
Skåne (R12)	1,377,827	Feb 25	March 02	March 04	March 03	March 12	March 18
Halland (R13)	333,848	Feb 27	March 08	March 09	March 01	March 18	March 24
Västra Götaland (R14)	1,725,881	Feb 27	Feb 26	Feb 28	Feb 26	Feb 28	March 13
Värmland (R17)	282,414	March 02	March 06	March 09	March 04	March 16	March 24
Örebro (R18)	304,805	March 09	March 04	March 08	March 04	March 13	March 30
Västmanland (R19)	275,845	Feb 29	March 13	March 16	March 13	March 16	March 28
Dalarna (R20)	287,966	March 03	March 11	March 17	March 12	March 17	March 22
Gävleborg (R21)	287,382	March 11	March 06	March 16	March 05	March 17	March 24
Västernorrland (R22)	245,347	March 11	March 10	March 13	March 15	March 23	March 27
Jämtland-Härjedalen (R23)	130,810	March 09	March 11	March 14	March 21	March 21	April 06
Västerbotten (R24)	271,736	March 10	March 10	March 14	March 11	March 18	April 06
Norrbotten (R25)	250,093	Feb 23	March 09	March 19	Feb 27	March 20	March 24
**Correlation coefficients [95% CI]**^2^	− 0.54	1	0.4 [− 0.09, 0.73]	0.27 [− 0.23, 0.65]	0.52 [0.07, 0.79]	0.39 [− 0.09, 0.72]	0.4 [− 0.09, 0.73]
**Median difference (IQR)**^3^		0	0.5 (− 1, 5.75)	7 (2.5, 10.5)	3.5 (− 0.75, 10.5)	13 (7.25, 16)	22 (15.25, 26.75)

^1^ A dash (“-”) in the ’First anomaly in call data’ column indicates that call data for this region is unavailable. ^2^ Pearson’s correlation coefficient, calculated between the dates of first detected anomalies in call data and each of the other reported dates in each region. ^3^ Median of the number of days between the first anomaly in call data and each corresponding event across regions.

**Figure 1:**
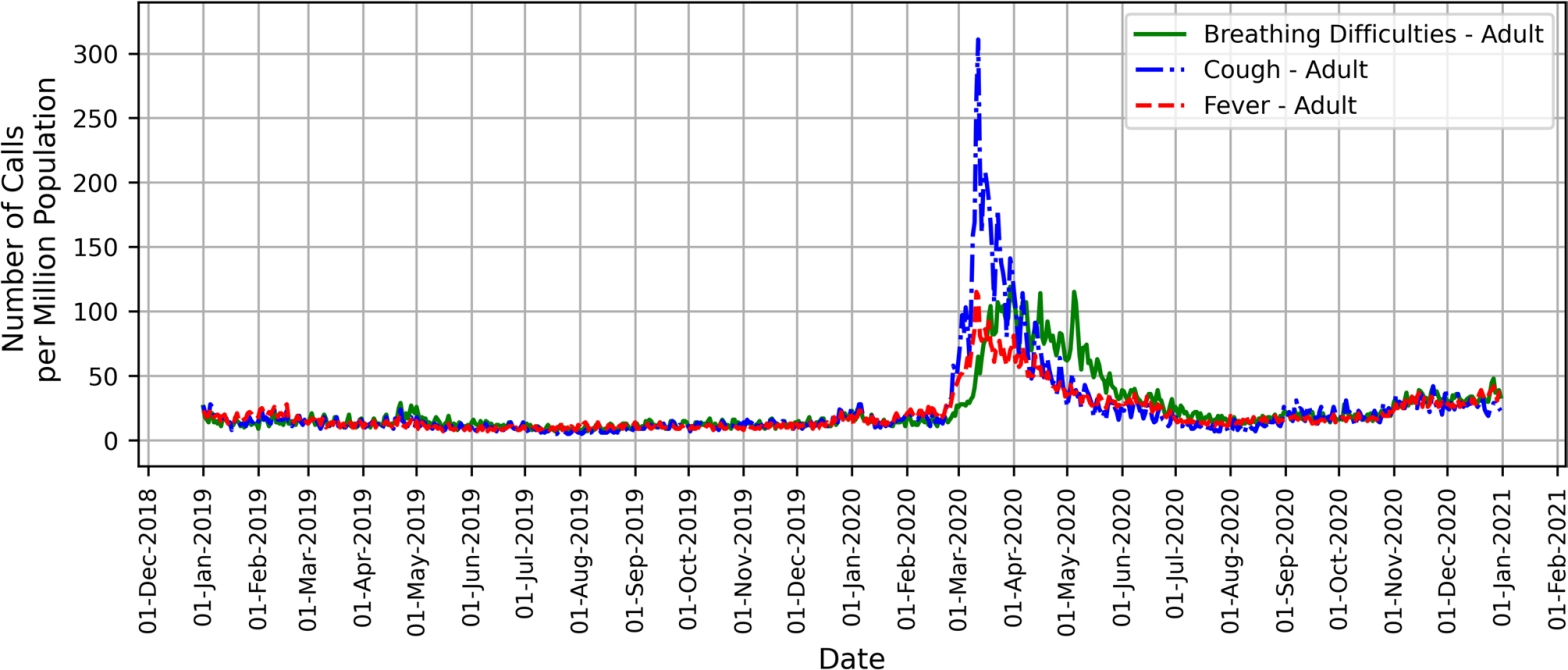
Daily call trends in Sweden for COVID-19 selected symptoms in 2019 and 2020.

## Results

### Data and trend analysis

Our analysis of the temporal trends in symptom-related calls to the 1177 helpline in Sweden includes the comparison of the daily call patterns for selected COVID 19 symptoms in 2019 and 2020. Figure 1 illustrates the daily call numbers per million population for COVID-19-related symptoms (breathing difficulties, cough, and fever), which shows a striking contrast between the years 2019 and 2020. In 2019, the number of calls with the selected symptoms remained almost stable throughout the year, however there is a notable spike in calls during the early weeks of the pandemic (weeks 11 to 14 for the first wave of the outbreak) in 2020. This surge corresponds with the onset of COVID-19 in Sweden (as well as with the influenza epidemic and human metapneumovirus) and illustrates significant shifts in symptom reporting patterns.

**Figure 2:**
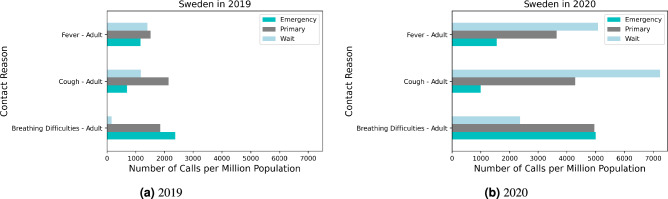
Distribution of calls in Sweden by referral priority (Emergency care vs. Primary care vs. Wait) for fever, cough, and breathing difficulties in (**a**) 2019 and (**b**) 2020.

### Referral priority analysis

**Figure 3:**
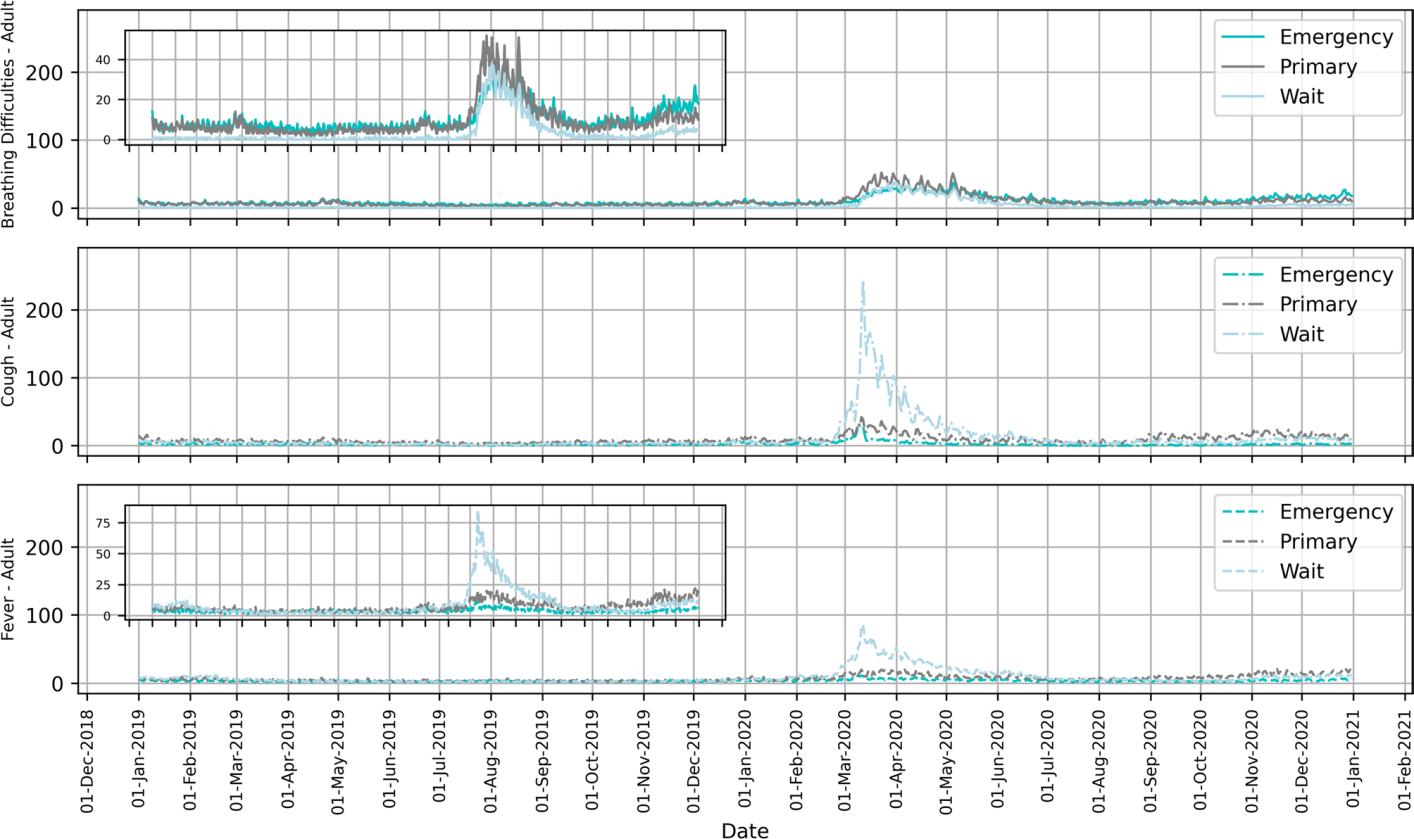
Daily call trends in Sweden for COVID-19 selected symptoms by referral priority in 2019 and 2020. The scale of the y-axis is per million population. For Fever and Breathing Difficulties, zoomed-in insets are included to highlight trends.

The frequency of the three referral priority categories Emergency, Primary care, and Waiting over the study period is shown in Fig. 2a,b. They show how urgency in healthcare-seeking behavior varied during the first year of the pandemic for the selected symptoms and highlights that the number of calls by referral priority shifted markedly for all three categories from 2019 to 2020. Figure 3 also shows the daily call numbers per million population categorized by referral priority for breathing difficulties, cough, and fever, highlighting an increase in waiting category calls for cough and fever among adults.

### Anomaly detection and correlation analysis

**Figure 4:**
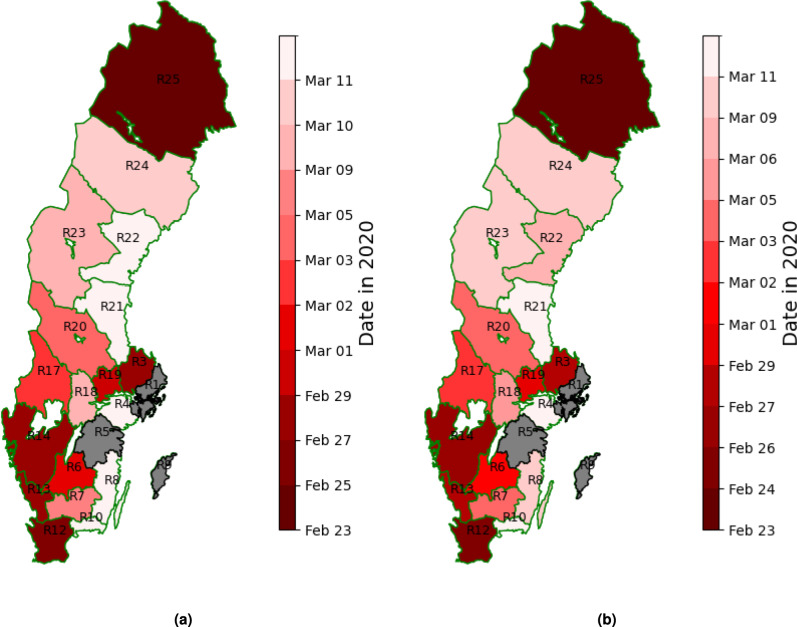
Geo-temporal visualization of first anomalies in daily call for COVID-19 selected symptoms in Sweden using (**a**) our anomaly detection algorithm with a threshold of 1.8, (**b**) z-score with a threshold of 4.

**Figure 5:**
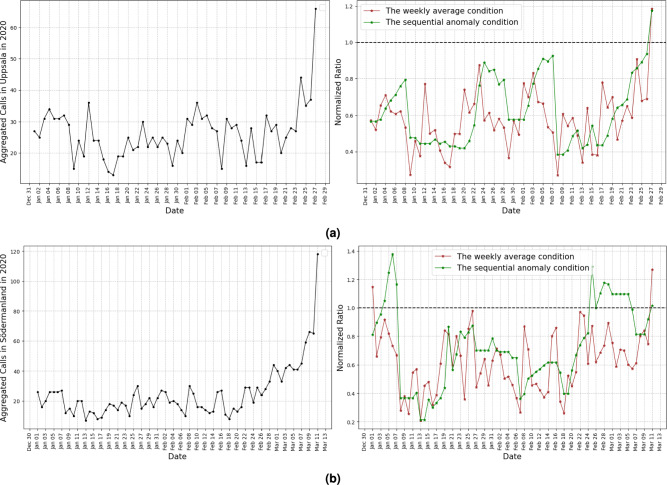
(**a**) Number of calls for COVID-19 selected symptoms and ratio values from our anomaly detection algorithm for the Uppsala region (R3), along with (**b**) number of calls for COVID-19 selected symptoms and ratio values for the Södermanland region (R4) as illustrative examples.

The COVID-19 outbreak reached different regions of Sweden at different time points between February 23, 2020, and March 11, 2020, according to the anomaly detection algorithm (Fig. 4a). Using the z-score method alone led to only marginal changes in the estimated outbreak pattern, which is illustrated in Fig. 4b. Figure 5a,b show the variable and the ratios from Equation (1) for Uppsala (R3) and Södermanland (R4), providing two examples of how our algorithm detects the first collective anomaly in the time series data in 2020.

**Figure 6:**
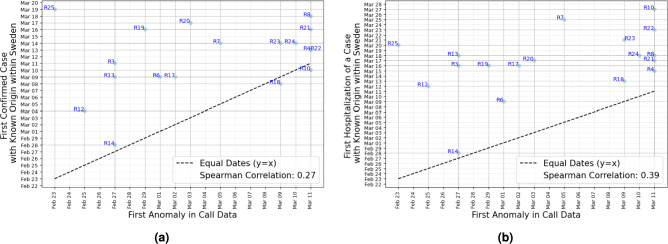
Correlation between the first anomaly in call data (Fig. 4a) with the date of (**a**) the first confirmed case infected in Sweden, and (**b**) the first hospitalized case infected in Sweden in 18 regions in 2020. The black dashed line represents the point of equality, used to compare whether anomalies in call data occur before or after events in each region.

The relationship between the timing of the first anomaly in call data and the date of the first confirmed and hospitalized cases infected in Sweden in each region in 2020 is illustrated in Fig. 6a,b. The scatter plot shows individual data points on the y-axis in relation to the corresponding first anomaly in the call data (on the x-axis). The black dashed line indicates where the values would be equal, serving as a reference to compare whether anomalies in call data occur before or after the events in each region. Spearman correlation coefficients further highlight the observed correlations. In certain regions, such as R25, anomalies were detected substantially earlier than the first confirmed COVID-19 case. While this may suggest a false positive, it is also plausible that these signals captured early, undetected transmission, particularly given the limited and delayed testing capacity during the initial phase of the pandemic.

**Figure 7:**
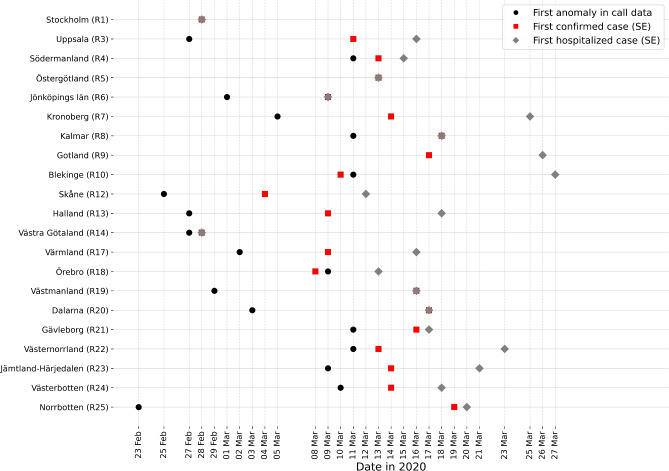
Timeline of early COVID-19 events across Swedish regions.

The key data for each region, including population, the date of the first detected anomaly in call data, the date of the first confirmed case, first confirmed case infected in Sweden, first hospitalized case, first hospitalized case infected in Sweden, and first death as well as the estimated dates of onset of the community spread and severe outcomes based on different data sources in this study are summarized in Table 2 and Appendix Table A1. They present the statistical analysis, including correlation coefficients with 95% confidence intervals and median differences with interquartile ranges, comparing the first detected anomalies in call data to other important dates. Almost exclusively, the first confirmed and hospitalized cases infected in Sweden, and the first death occurred later than anomalies detected in the 1177 helpline data, with medians of 7 days (IQR: 2.5, 10.5), 13 days (IQR: 7.25, 16), and 22 days (IQR: 15.25, 26.75), respectively. Figure 7 also provides a visual timeline of early COVID-19 events across Swedish regions, complementing the data in Table 2 and aiding regional comparison.

As can be seen in Table 2, for both infections and hospitalizations, the anomaly signal correlated stronger with the first case than the first case of Swedish origin. This pattern is expected, as people usually call soon after the onset of the symptom, regardless of the origin of the infection. Regarding community spread (Appendix Table A1), the anomaly signal aligns more closely with absolute thresholds for community spread than with relative thresholds, and it shows stronger correlations with confirmed cases and hospitalizations than with deaths. Furthermore, it should be noted that, the strongest correlations observed in Tables 2 and A1 are of similar magnitude, supporting the robustness of the anomaly signal as an early proxy for emerging community spread for both sources of validation data.

Standard ROC analysis is not directly applicable in our setting, not only due to the absence of a well-defined ground truth, but also because we are estimating a time point and a time difference (in days) rather than a binary outcome. Instead, the reported IQR of the time difference versus the anomaly signal can be used to describe the variability in prediction error across regions. IQR thus serves as an indication of how well the anomaly signals in call data could have predicted the occurrence of COVID-19-related events across regions.

For instance, in Table 2 under the column for ”first hospitalized case infected in Sweden”, the median time difference suggests that we can predict that the first case infected in Sweden requiring hospitalization would occur 13 days (median time difference) after the anomaly signal. Additionally, we can interpret the IQR (7.25 to 16) as the variability in the prediction error across regions. This suggests that in 50% of regions, the actual event (hospitalization) occurred between -6 (6 days earlier) and +3 (3 days later) than predicted, relative to the anomaly detection date.

To assess the impact of seasonal effects, we performed a complementary analysis as mentioned in the method development part. Two regions were excluded due to a lack of data. For the remaining 16 regions with sufficient historical data, the adjustment yielded largely consistent anomaly detection dates, with only one region (Uppsala, R3) showing a one-day difference in the first detected anomaly.

The estimated onset of community spread, based on relative and absolute confirmed cases, as well as the onset of severe outcomes based on relative and absolute hospitalizations and deaths, occurs considerably later than when anomalies in helpline calls are used (Appendix Figure A1).

To enhance the contextual understanding of the data, we have included a supplementary table (Appendix Table A2) presenting the average daily number of calls per region related to selected COVID-19 symptoms (cough, fever, and breathing difficulties) in 2019 and 2020. The table also reports regional disposable income for the same years as a proxy for socioeconomic status. The data indicate that average disposable income varies only modestly across regions, suggesting limited regional socioeconomic disparities. In the Swedish context, socio-economic differences are mainly observed within different parts of the regions and even within cities while regions themselves are relatively comparable.

## Discussion

The findings of the present study highlight the potential of monitoring 1177 helpline calls as an early indicator of infectious disease outbreaks. According to our proposed monitoring algorithm, the emerging COVID-19 pandemic in 2020 led to a surge in influenza-like symptoms at different time points in different regions in Sweden, starting on February 23, 2020. This surge was present in all included regions 17 days later, on March 11, 2020. On the regional level, the algorithm was able to signal community spread on average one week earlier than the first officially confirmed case of domestic origin and almost two weeks earlier on average than a domestic case was hospitalized. This finding aligns with a recent study by Dyrdak et al.^41^, who used stored respiratory samples and genome sequencing, and found that sustained community transmission in Sweden began at least a week earlier than previously recognized.

A major strength of the present study was that the available data, covering 18 out of 21 Swedish regions and 72% of the population, comes from a nationally implemented telehealth system (a service that is generally accessible to the public at no cost). Another strength was the granularity in the available individual-level data on symptoms, referral priority, confirmed cases, their likely origin, hospitalizations and deaths, which facilitated the estimation of spatiotemporal differences in disease transmission dynamics.

A limitation with the use of call data for anomaly detection is that citizens tend to use telehealth services more frequently in the event of a health crisis, such as a pandemic^42^. This would make the anomalies in call data more prone to noise and thereby dilute the association with the actual spread of the disease in society. Additionally, the absence of detailed data on media coverage and public announcements limits our ability to distinguish between increased calls due to awareness versus actual disease spread. Moreover, some studies have shown that clinical cases tend to represent a more consistent proportion of total cases compared to those reported through self-reported digital apps^43^. Specifically, clinical cases demonstrated the highest correlation with unbiased household survey data, highlighting their reliability as a timely epidemic indicator. In contrast, self-reported data from digital apps, while also strongly correlated, captured a less consistent proportion of cases. Another important limitation was that the call data from the largest region (Stockholm) was not available, especially since this region was severely and earlier affected by the first wave of the pandemic^44^.

Still, syndromic surveillance can be a helpful tool in detecting, monitoring, and managing public health events of concern^45^. For example, the NHS Direct syndromic surveillance system has played a pivotal role in detecting and evaluating the impact of various health crises in the UK, including the 2009 influenza pandemic, the widespread flooding in 2007, and the ash cloud resulting from the Eyjafjallajökull volcanic eruption in Iceland in 2010^11^. Data from the 1177 helpline holds promise for syndromic surveillance in Sweden^24–27^, such as through time series anomaly detection (TAD) that can enable timely and accurate detection in surveillance systems. While several TAD methods have been proposed^46,47^, the lack of a universally accepted definition of anomalies, along with challenges like dynamic changes in normal behavior, and the difficulty of generalizing across different domains, pose significant obstacles^47,48^. Implementing data-driven approaches offers a promising solution to these challenges. The anomaly detection algorithm in this paper is a data-driven approach designed to identify both collective and point-wise anomalies. The z-score method produces results similar to our algorithm, which is expected in the context of COVID-19. However, we have tailored the more complex criteria to detect sequential outlier patterns. One should also consider that an important strength of anomaly detection approaches is their applicability to real-time surveillance. Although the present study used historical data, the analytical process was designed for prospective application, continuously assessing each new day’s data against recent trends using predefined rules to enable dynamic, real-time monitoring.

Variations in healthcare-seeking behavior and telehealth accessibility, shaped by factors such as demographics, healthcare infrastructure, and regional policies, can also influence the performance of anomaly detection. In this study, we used regional disposable income as a proxy for socioeconomic deprivation and observed relatively limited variability across Swedish regions. However, in settings with greater socioeconomic heterogeneity, incorporating deprivation indices would be crucial to assess and ensure the robustness of the anomaly detection method across diverse population groups. To account for such variation, the algorithm could be adapted in future applications by stratifying the data and computing thresholds separately for different socioeconomic strata or by including deprivation indices as covariates in a hierarchical modeling framework. These modifications would help the method adjust for structural differences in baseline call behavior related to access to care, risk perception, or health literacy.

Our findings suggest that COVID-19 was likely seeded at multiple locations in Sweden around the same time, but nevertheless escalated at different speeds across regions. This escalation exhibited a clear inverse association with population size, where our algorithm signaled anomalies earlier in more populated regions. This may reflect a larger inflow of the virus in those regions from abroad initially^49^, but may also reflect the importance of population density as a determinant of the spread of emerging diseases^50^. The observed pattern, where various areas experienced seeding simultaneously but with transmission escalating at different rates, is difficult to capture with diffusion models for spatio-temporal analyses^51^, which assume a more uniform spread. While methods like genome sequencing are also valuable for tracking the virus^52^, approaches such as ours based on helpline call data, offer a solution that provides real-time insights into the spatiotemporal spread, making it a cost-effective means for early detection of community transmission.

The Swedish Public Health Agency communicated on March 4, 2020, that all confirmed cases in Sweden were still linked to travelling^53^. Clear signs of community spread were first officially observed on March 10 in the two largest regions, Stockholm and Västra Götaland, but still no general spread was noted for the rest of the country^54^. For comparison, our monitoring algorithm had signaled symptom anomalies in 9 regions by March 4, in 13 regions by March 10, and in the remaining 5 regions the next day. The delay in the official reporting of community spread was most likely due to the limited testing for SARS-CoV-2, which during the early phase of the pandemic in Sweden was restricted to suspected cases linked to travelling and to people with symptoms seeking hospital care. A less restrictive testing policy could have led to earlier identification of domestic origin cases. However, our findings suggest that syndromic surveillance could have monitored disease transmission and detected community spread earlier, even without extensive testing.

The present study was part of the larger SWECOV project (Swedish Register-based Research Program on COVID-19), with cross-linked register data for the full population of Sweden during the pandemic years^55^. This data infrastructure holds many promises for additional research. Future studies in this setting could, for example, increase the spatio-temporal granularity down to neighborhood or city areas, incorporate individual-level data on socioeconomic conditions, comorbidities, and health outcomes, integrate advanced machine learning models into disease surveillance, and include additional validation of our signal against other indicators of community spread, such as^41^. In conclusion, health helpline data can enhance public health surveillance by enabling early outbreak detection and monitoring spatio-temporal disease transmission. It is especially valuable in resource-limited settings for planning healthcare needs, protecting risk groups, and limiting disease spread.

## Supplementary Information

Supplementary Information.

## Data Availability

While some of the datasets analyzed in this study are publicly available, the telehealth call data remains restricted due to ethical considerations under the SWECOV project. Researchers interested in access may contact the SWECOV project coordinator for additional information. Dominik Dietler should be contacted if someone wants to request the data from this study.
