# Supplementary material for: Time series anomaly detection in helpline call trends for early detection of COVID-19 spread across Sweden, 2020
Source: Sci Rep. 2025 Sep 24;15:32701. doi: 10.1038/s41598-025-20641-2 (PMC12460667; doi:10.1038/s41598-025-20641-2)
Supplement: Supplementary file 1 — Supplementary Information. [file 41598_2025_20641_MOESM1_ESM.pdf]

Table A1. Summary of important dates and statistical analysis of first detected anomalies in call data compared to estimated dates of community spread and severe outcomes in Sweden (2020).

Region	First anomaly in call data [1]	RT[2]	Comm. Spread (Rel)[3]	Comm. Spread (Abs)[4]	Severe Outcome (Hosp. Rel)[5]	Severe Outcome (Hosp. Abs)[6]	Severe Outcome (Death Rel)[7]	Severe Outcome (Death Abs)[8]
Stockholm (R1)	-	594	March 20	March 06	March 23	March 14	April 09	March 26
Uppsala (R3)	Feb 27	96	March 25	March 22	April 03	March 31	April 26	April 18
Södermanland (R4)	March 11	74	March 24	March 24	March 24	March 24	April 05	April 05
Östergötland (R5)	-	116	March 20	March 18	March 28	March 25	April 25	April 15
Jönköping (R6)	March 01	91	March 27	March 26	April 02	April 01	May 05	April 29
Kronoberg (R7)	March 05	50	April 04	April 08	April 21	April 30	May 20	June 10
Kalmar (R8)	March 11	61	April 07	April 13	April 08	April 12	June 28	Nov 10
Gotland (R9)	-	15	March 25	May 30	May 02	Nov 26	Nov 19	-
Blekinge (R10)	March 11	40	April 10	May 05	June 03	July 05	Dec 27	-
Skåne (R12)	Feb 25	344	April 05	March 11	April 20	March 29	Nov 16	May 01
Halland (R13)	Feb 27	83	March 28	March 26	April 15	April 12	July 13	June 20
Västra Götaland (R14)	Feb 27	431	April 01	March 11	April 07	March 20	May 09	April 11
Värmland (R17)	March 02	71	April 10	April 12	April 20	April 21	July 07	Oct 13
Örebro (R18)	March 09	76	March 27	March 27	April 04	April 04	May 05	May 05
Västmanland (R19)	Feb 29	69	March 30	March 31	April 01	April 01	April 17	April 18
Dalarna (R20)	March 03	72	March 27	March 27	March 28	March 28	April 13	April 13
Gävleborg (R21)	March 11	72	March 29	March 29	April 03	April 04	April 30	May 02
Västernorrland (R22)	March 11	61	April 04	April 07	April 12	April 15	May 11	May 23
Jämtland-Härjedalen (R23)	March 09	33	March 21	March 28	April 13	May 07	May 31	Dec 11
Västerbotten (R24)	March 10	68	April 02	April 02	April 15	April 18	Dec 14	Dec 25
Norrbotten (R25)	Feb 23	63	April 01	April 03	April 02	April 04	June 15	July 07
**Correlation Coeff. [95% CI]**[9]			0.09 [-0.39, 0.54]	0.49 [0.03, 0.78]	0.08 [-0.40, 0.53]	0.4 [-0.08, 0.73]	-0.04 [-0.5, 0.43]	0.19 [-0.32, 0.62]
**Median difference (IQR)**[10]			27 [23.25,30]	24.5 [18.25,32.5]	35.5 [29,45.25]	33 [27.25,44]	74 [57.5,123.5]	66 [51,135]

[1] A dash ("-") in the 'First anomaly in call data' column indicates that call data for this region are unavailable.
[2] Relative Threshold.
[3] Start of Community Spread Based on Confirmed Cases (Relative).
[4] Start of Community Spread Based on Confirmed Cases (Absolute).
[5] Start of Severe Outcome Based on Hospitalization (Relative).
[6] Start of Severe Outcome Based on Hospitalization (Absolute).
[7] Start of Severe Outcome Based on Death (Relative).
[8] Start of Severe Outcome Based on Death (Absolute).
[9] Pearson's correlation coefficient, calculated between the dates of first detected anomalies in call data and each of the other reported dates in each region.
[10] Median of the number of days between the first anomaly in call data and each corresponding event across regions.

Table A2. Average daily number of calls (per 100k) for selected COVID-19-related symptoms and disposable income in Sweden's regions in 2019 and 2020. Disposable income is reported in thousands of Swedish kronor (SEK).

Region	Avg. Daily Calls (COVID-19 Symptoms) per 100k (2019)	Avg. Daily Calls (COVID-19 Symptoms) per 100k (2020)	Disposable Income 2019	Disposable Income 2020
Stockholm (R1)	-	-	360	365
Uppsala (R3)	5.22	13.55	399	404
Södermanland (R4)	-	12.43	361	366
Östergötland (R5)	-	-	340	345
Jönköpings län (R6)	4.40	9.35	346	352
Kronoberg (R7)	4.96	12.41	360	364
Kalmar (R8)	4.48	8.96	344	348
Gotland (R9)	-	-	336	341
Blekinge (R10)	5.64	10.65	321	328
Skåne (R12)	2.32	8.35	335	342
Halland (R13)	2.99	8.39	339	347
Västra Götaland (R14)	3.53	10.61	368	373
Värmland (R17)	-	10.98	366	370
Örebro (R18)	5.25	12.46	341	343
Västmanland (R19)	5.08	12.33	341	347
Dalarna (R20)	5.21	9.72	348	354
Gävleborg (R21)	5.57	11.83	340	346
Västernorrland (R22)	5.71	12.23	335	340
Jämtland-Härjedalen (R23)	5.35	13.76	349	355
Västerbotten (R24)	5.52	11.04	336	342
Norrbotten (R25)	4.00	10.00	348	353

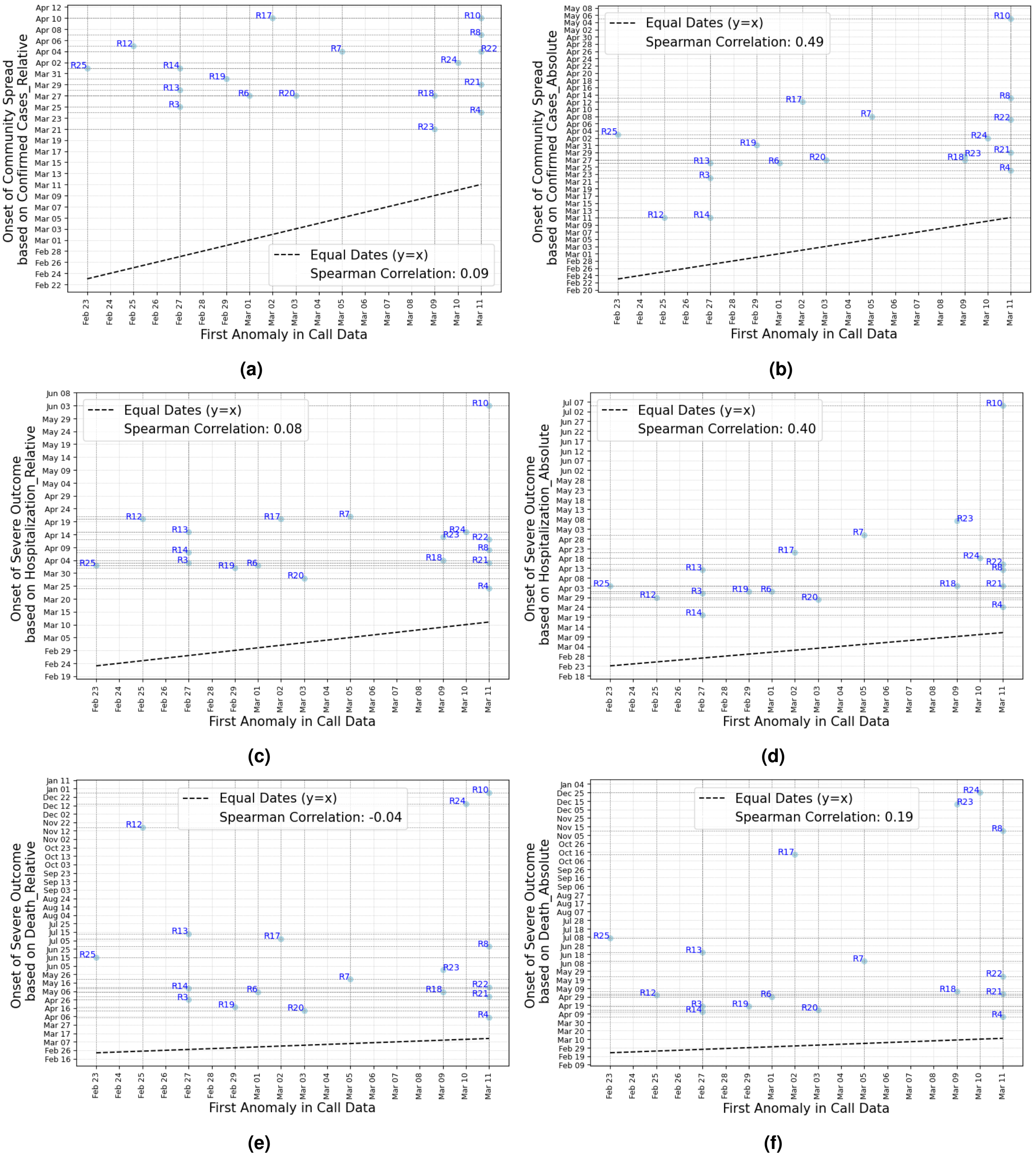

Figure A1. Correlation between the first anomaly in call data (Figure 4a) with the date of a) the onset of community spread based on confirmed cases (relative threshold), b) the onset of community spread based on confirmed cases (absolute threshold), c) the onset of severe outcome based on hospitalization (relative threshold), d) the onset of severe outcome based on hospitalization (absolute threshold), e) the onset of severe outcome based on death (relative threshold), and f) the onset of severe outcome based on death (absolute threshold) in 18 regions in Sweden in 2020.
